# Modifiable Variables Are Major Risk Factors for Posttransplant Diabetes Mellitus in a Time-Dependent Manner in Kidney Transplant: An Observational Cohort Study

**DOI:** 10.1155/2020/1938703

**Published:** 2020-03-18

**Authors:** Débora Dias de Lucena, João Roberto de Sá, José O. Medina-Pestana, Érika Bevilaqua Rangel

**Affiliations:** ^1^Nephrology Division, Universidade Federal de São Paulo/Hospital do Rim, São Paulo, SP, Brazil; ^2^Endocrinology Division, Universidade Federal de São Paulo, São Paulo, SP, Brazil; ^3^Hospital Israelita Albert Einstein, São Paulo, SP, Brazil

## Abstract

Modifiable and nonmodifiable risk factors for developing posttransplant diabetes mellitus (PTDM) have already been established in kidney transplant setting and impact adversely both patient and allograft survival. We analysed 450 recipients of living and deceased donor kidney transplants using current immunosuppressive regimen in the modern era and verified PTDM prevalence and risk factors over three-year posttransplant. Tacrolimus (85%), prednisone (100%), and mycophenolate (53%) were the main immunosuppressive regimen. Sixty-one recipients (13.5%) developed PTDM and remained in this condition throughout the study, whereas 74 (16.5%) recipients developed altered fasting glucose over time. Univariate analyses demonstrated that recipient age (46.2 ± 1.3*vs*. 40.7 ± 0.6 years old, OR 1.04; *P* = 0.001) and pretransplant hyperglycaemia and BMI ≥ 25 kg/m^2^ (32.8% *vs*. 21.6%, OR 0.54; *P* = 0.032 and 57.4% *vs*. 27.7%, OR 3.5; *P* < 0.0001, respectively) were the pretransplant variables associated with PTDM. Posttransplant transient hyperglycaemia (86.8%. 18.5%, OR 0.03; *P* = 0.0001), acute rejection (*P* = 0.021), calcium channel blockers (*P* = 0.014), TG/HDL (triglyceride/high-density lipoprotein cholesterol) ratio ≥ 3.5 at 1 year (*P* = 0.01) and at 3 years (*P* = 0.0001), and tacrolimus trough levels at months 1, 3, and 6 were equally predictors of PTDM. In multivariate analyses, pretransplant hyperglycaemia (*P* = 0.035), pretransplant BMI ≥ 25 kg/m^2^ (*P* = 0.0001), posttransplant transient hyperglycaemia (*P* = 0.0001), and TG/HDL ratio ≥ 3.5 at 3-year posttransplant (*P* = 0.003) were associated with PTDM diagnosis and maintenance over time. Early identification of risk factors associated with increased insulin resistance and decreased insulin secretion, such as pretransplant hyperglycaemia and overweight, posttransplant transient hyperglycaemia, tacrolimus trough levels, and TG/HDL ratio may be useful for risk stratification of patients to determine appropriate strategies to reduce PTDM.

## 1. Introduction

Posttransplant diabetes mellitus (PTDM) develops in 10-20% of patients after kidney transplant and is a major risk factor for cardiovascular disease and death [1]. PTDM adversely affects graft survival and increases medical costs as well [2, 3].

PTDM results from predisposing factors, similarly to type 2 diabetes mellitus (DM), but also because of specific posttransplant risk factors. However, microvascular complications of PTDM diagnosed more than five years seem to be milder than expected for type 1 and type 2 DM [4].

Major risk factors for development of PTDM are metabolic adverse effects of immunosuppressive regimen, including calcineurin inhibitors, mammalian target of rapamycin inhibitors (mTORi), and corticosteroids, posttransplant viral infections, and hypomagnesaemia, in addition to the traditional risk factors seen in patients with type 2 DM [5–8]. Therefore, modifiable and nonmodifiable variables are risk factors for PTDM. Modifiable risk factors include insufficient physical activity, metabolic syndrome, hepatitis C virus, cytomegalovirus (CMV), and immunosuppressive regimen. Nonmodifiable risk factors include age, family history of DM, autosomal-dominant polycystic kidney disease (ADPKD), African-American and Hispanic ethnicities, and some human leukocyte antigen (HLA) genotypes. Other factors such as body mass index (BMI), biopsy-proven acute rejection, initial graft function, proteinuria, and thiazide diuretics were also associated with the risk of PTDM [5, 6, 9].

Early identification of patients at risk of PTDM may lead ultimately to risk stratification of patients to determine appropriate strategies to reduce the occurrence of PTDM, including lifestyle modification and pharmacological treatment [9–12].

Here, we aimed to verify the prevalence of PTDM and its risk factors in a single-center cohort study comprising recipients under current immunosuppressive regimen in the modern era. Our study included analyses of modifiable or nonmodifiable variables over three years after kidney transplant. We documented that recipient age was the only nonmodifiable variable that contributed to PTDM, whereas modifiable variables were the major risk factors before and after transplant. Notably, transient hyperglycaemia and triglyceride/high-density lipoprotein cholesterol (TG/HDL) ratio may be useful tools for the identification of insulin metabolism impairment behind PTDM.

## 2. Patients and Methods

### 2.1. Subjects

The study protocol was approved by the Research Ethics Committee of the Federal University of São Paulo, Brazil (protocol number 66288217.0.0000.5505), and included 450 deceased or living kidney recipients on 932 adult recipients who were transplanted at Hospital do Rim e Hipertensão, São Paulo, SP, Brazil, over one-year period from January 1, 2011 to December 31, 2011. All subjects were followed-up for at least three years, except those who died (*n* = 21) or exhibited graft loss (*n* = 15) before the end of the study. Because we investigated PTDM, 104 patients with DM before transplant were excluded, as well as simultaneous pancreas-kidney transplant (*n* = 49), pancreas after kidney transplant (*n* = 10), recipients under 18 years old (*n* = 41), recipients who were transferred to other hospital (*n* = 238), and retransplant (*n* = 4). Medical records were then retrospectively analysed, and the number of 450 recipients was defined by statistical power sampling.

### 2.2. Immunosuppression

All patients received methylprednisolone 1.0 g during the intraoperative period. Immunosuppressive maintenance regimen was based on tacrolimus (FK), prednisone (PRED), and sodium mycophenolate (MPS) in deceased donor (DD) recipients with panel reactive antibody (PRA) ≥ 50% or PRA < 50% plus expanded criterion donors. For these patients, we prescribed thymoglobulin induction 1 mg/kg/day (ranging from three to six doses). For PRA < 50% with standard donors, FK, PRED, and azathioprine (AZA) or FK, PRED, and mammalian target of rapamycin inhibitors (mTORi) regimens were used. For living donor (LD) recipients with low immunological risk (PRA < 50% and first transplant) and identical HLA, the immunosuppressive regimen was based on cyclosporine (CsA), PRED, and AZA.

Immunosuppressive regimen was based on FK-PRED-MPS (*n* = 215), FK-PRED-AZA (*n* = 146), FK-PRED-everolimus (*n* = 20), FK-PRED-sirolimus (*n* = 2), CsA-PRED-MPS (*n* = 35), sirolimus-PRED-AZA (*n* = 4), everolimus-PRED-MPS (*n* = 8), and sirolimus-PRED-MPS (*n* = 8). There are also those who were using only two drugs due to severe adverse event after transplant, e.g., MPS-PRED (*n* = 3), sirolimus-PRED (*n* = 1), and everolimus-PRED (*n* = 2). All regimens included prednisone. The wide variety of immunosuppressive regimens is due to the start of testing of some protocols in the period.

Initial dose of FK was 0.1 mg/kg/dose twice daily and trough level adjusted to 5-15 ng/mL in association with AZA or MPS and adjusted to 3-5 ng/mL when combined with mTORi. MPS started at a dose of 1440 mg/day. We used AZA at a dose of 2 mg/kg/day and everolimus at a dose of 3 mg/day, with subsequent trough level adjustment for 4-8 ng/mL. For patients receiving sirolimus, we used a first-dose single dose of 6 mg and then 2-5 mg/day in a single daily dose to maintain trough level of 5-10 ng/mL. We started PRED at 30 mg/day, followed by dose reduction up to 5 mg/day within 1-6 months after transplant. CsA therapy started at a dose of 3-6 mg/kg twice daily with trough level of 100-300 ng/mL.

### 2.3. Definition of PTDM

Criteria outlined in the 2003 international consensus guidelines and 2014 updated recommendations defined PTDM [9, 13]. Patients with a fasting plasma glucose level ≥ 126 mg/dL or symptoms of diabetes (polyuria, polydipsia, and unexplained weight loss) plus random plasma glucose ≥ 200 mg/dL, confirmed on a subsequent day or requiring prolonged (30 days) treatment with insulin or oral hypoglycaemic agent were identified as PTDM (“modified” American Diabetes Association). Fasting blood glucose was measured once a week in the first month posttransplant, weekly to twice a month in the first three-month posttransplant, monthly up to one-year posttransplant and then every two to three months.

### 2.4. Risk Factors

Nontransplant variables included age, gender, ethnicity, chronic kidney disease (CKD) etiology, mean time on dialysis, pretransplant body mass index (BMI) and hyperglycaemia, cytomegalovirus (CMV) serology, and human leukocyte antigen (HLA) genotypes. Transplant-related variables included acute rejection, cumulative steroid dose, immunosuppressive regimen, CMV infection, transient hyperglycaemia, delayed graft function, hypertension, antihypertensive drugs, triglyceride/high-density lipoprotein cholesterol (TG/HDL) ratio, and FK trough level.

Weight in kilograms divided by height in meters squared defined BMI. Delayed graft function was defined by dialysis requirement during the first week posttransplant. Transient hyperglycaemia defined by two measurements of altered fasting glucose level ≥ 126 mg/dL or random plasma glucose ≥ 200 mg/dL within three months of transplant, whereas three months after transplant defined PTDM diagnosis. Cumulative dose of corticosteroids was calculated by prednisone dose, in milligrams (mg), within the first six months after transplant and by methylprednisolone dose during perioperative period and pulse treatment for acute rejection (dose 1 g/day for 3-5 days depending on acute rejection grade), and then adjusted by the body weight in kilograms (kg). Acute rejection included the increase in serum creatinine without any other apparent cause and/or by allograft biopsy. To assess triglyceride/high-density lipoprotein cholesterol (TG/HDL) ratio, a value greater than 3.5 identified patients who were under high cardiovascular risk [14].

Estimated glomerular filtration rate (eGFR) was calculated within 1, 3, 6, 12, 24, and 36 months after kidney transplant by Chronic Kidney Disease Epidemiology Collaboration (CKD-EPI) formula: 175 × serum creatinine − 1.154 × age − 0.203 × 1.212 (if black) × 0.742 (if female), where the eGFR is expressed in mL/min/1.73 m^2^ body surface area [15].

### 2.5. Statistical Analysis

To determine which modifiable and nonmodifiable risk factors, either transplant-related or nontransplant-related, were associated with PTDM development, we divided patients into 2 groups: PTDM present or PTDM (+) and PTDM absent or PTDM (-). All putative factors that were univariately associated at *P* ≤ 0.1 entered in a linear regression model with PTDM as the dependent variable for further multivariate analyses. Thus, we divided the variables into demographic (recipient age, pretransplant hyperglycaemia, and pretransplant BMI ≥ 25 kg/m^2^), related to transplant (FK trough level at one, three, and six months and acute rejection), and cardio-metabolic (transient hyperglycaemia and TG/HDL ratio ≥ 3.5) variables. Results were expressed as odds ratio (OR) with 95% confidence interval (CI). Kaplan-Meier curve was used to evaluate the cumulative incidence of PTDM. Values of *P* < 0.05 were considered statistically significant. We used Statistical Product and Services Solutions, version 18.0, SPSS Inc., Chicago, IL, USA, and GraphPad Prism (version 7.0, San Diego, CA, USA).

## 3. Results

**Figure 1:**
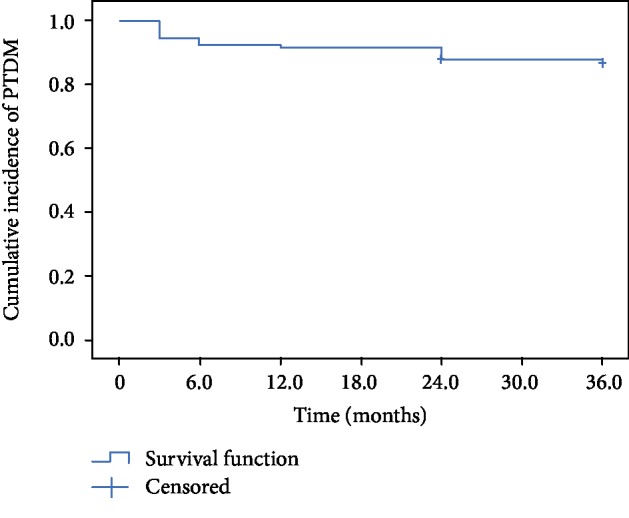
Kaplan-Meier curve showing the incidence of PTDM over time.

Table 1 describes demographic characteristics. PTDM was diagnosed in 61 patients (13.5%), while 315 (70%) remained with normal blood glucose levels, and 74 (16.5%) developed altered fasting glucose (Figure 1).

By univariate analyses, major risk factors for PTDM development included nontransplant factors, either modifiable or nonmodifiable factors, such as recipient age and pretransplant hyperglycaemia and pretransplant BMI classified as overweight and obesity (Table 2). Transplant-related risk factors for PTDM comprised renal allograft rejection, transient hyperglycaemia, calcium channel blockers, as well as TG/HDL ratio ≥ 3.5 at 1 year and 3 years and FK trough levels at 1, 3, and 6 months after transplant (Table 3). However, the cumulative dose of corticosteroids within six months after transplant was not a risk factor for PTDM. We selected the most common HLA genotypes in our study population, yet we did not identify any HLA genotype that was associated with PTDM.

By multivariate analyses, pretransplant hyperglycaemia and pretransplant BMI ≥ 25 kg/m^2^ resulted in increased risk of PTDM (Table 4). Likewise, transplant-related variables, such as transient hyperglycaemia within the first three months and TG/HDL ≥ 3.5 at 3 years after transplant, contributed to PTDM. The latter may at least in part explain why insulin resistance supported PTDM diagnosis over time.

PTDM did not have impact on eGFR values in the PTDM (+) group of both LD and DD recipients during any time-point up to 36 months after transplant (*P* = 0.26) ([Supplementary-material supplementary-material-1]). However, in the PTDM (-) group, LD recipients had a higher eGFR when compared to DD recipients until 24 months after transplant (~10 mL/min/1.73m^2^), so that in the first month after transplant that difference was more pronounced (~20 mL/min/1.73m^2^, *P* < 0.0001) ([Supplementary-material supplementary-material-1]).

In light of donor type, we verified that LD recipients exhibited lower eGFR values within 36 months when compared to the first month after transplant in the PTDM (-) group (*P* = 0.007) ([Supplementary-material supplementary-material-1]), while LD recipients in the PTDM (+) group did not present difference over time. Among DD recipients, eGFR was higher over time until 24 months after transplant in the PTDM (-) group (*P* < 0.0001). In DD recipients who developed PTDM, eGFR was not affected over time ([Supplementary-material supplementary-material-1]). To note, the lack of impact of PTDM on eGFR may be attributed to the short follow-up in our study.

**Figure 2:**
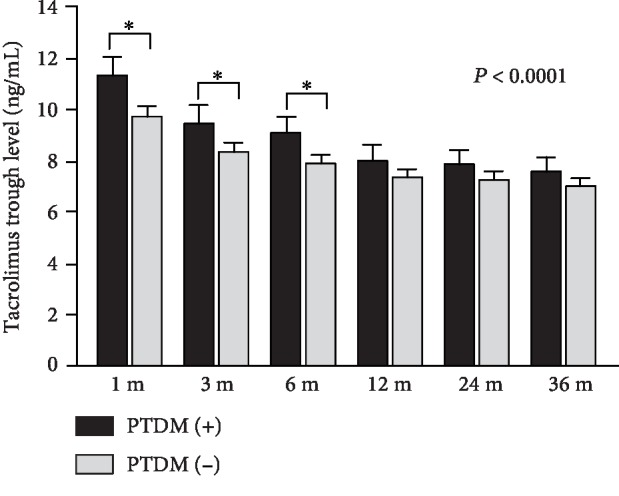
Tacrolimus (FK) trough level over time in months (m) in the PTDM (+) and PTDM (-) groups.

**Figure 3:**
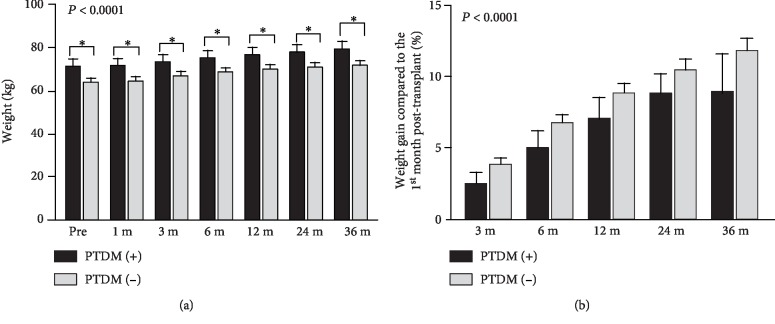
(a) Weight analysis over time in months (m) in the PTDM (+) and PTDM (-) groups. (b) Weight gain throughout the follow-up in comparison to the first month posttransplant in the PTDM (+) and PTDM (-) groups. PTDM (+) and PTDM (-) groups were not different, although both groups exhibited weight gain over time.

Of importance, metabolic adverse effects of immunosuppressive regimen included higher FK trough levels at 1, 3, and 6 months in the PTDM (+) group (Figure 2), pointing out the importance of tailoring FK dose for PTDM prevention. Likewise, the PTDM (+) group exhibited higher values of weight over all time-points (Figure 3(a)). To note, that difference was documented even before the transplant, which highlights the benefit of early intervention in lifestyle modification. On top of that, in all groups, either PTDM (-) or PTDM (+), we verified weight gain over time when compared to the first month after transplant (*P* < 0.0001; Figure 3(b)). However, we emphasize that the difference in weight gain between the groups was not statistically significant.

Conversely, cumulative doses of corticosteroids within the first six months after, a well-known risk factor for weight gain, were not different between PTDM (+) and PTDM (-) groups (70.3 ± 7.7 mg/kg versus 61.8 ± 1.8 mg/kg, respectively, *P* = 0.2), yet those doses were slightly higher in the former group (Table 3).

## 4. Discussion

Our study documented a PTDM cumulative incidence within three-year posttransplant of 13.5% in recipients using current immunosuppressive regimen in the modern era, which is in agreement with rates of 7.5-21% in the literature [16, 17]. PTDM rates may increase over time, e.g., 27%, 21%, 21%, and 30% within 3, 12, 24, and 36 months after transplant, respectively [16]. To note, most of the cases (83.7%) occur in the first year after transplant [18]. PTDM results from predisposing factors that are similar to type 2 DM [19], but also because of specific posttransplant risk factors. Although PTDM has many characteristics in common with type 2 DM, the prevention and treatment of the two disorders are often different.

Therefore, the risk of developing DM increases with age, as aging is associated with insulin resistance and reduced *β*-cell function [19]. In kidney transplant setting, age greater than 45 years old increases PTDM risk 2.2 times when compared to individuals of 18-44 years old [20], which is in accordance to our findings. Likewise, gender may increase the risk of PTDM in a different manner, so that females may present a two-fold increase in BMI when compared to males, which put them at a greater risk [21]. Abdominal circumference greater than 94 cm may predict PTDM in males, whereas the major risk factor for PTDM was BMI > 30 kg/m^2^ in women [2, 22]. Ethnicity contributes equally to increase the risk of PTDM, yet either Hispanic and Caucasian individuals or African-American individuals may be at risk [20, 23]. In our study, we did not find an association of PTDM and ethnicity, which may be explained by the fact that Brazilian population is highly mixed.

Pretransplant metabolic changes, such as the increase in BMI and glucose metabolism impairment, are major risk for PTDM [5]. Pretransplant hyperglycaemia indicates that insulin resistance or insulin secretion deficiency has already been present for an unknown period and may ultimately contribute to PTDM [24]. Some HLA genotypes may also result in PTDM, such as HLA-B27, HLA-DR3, and HLA-A3 [25, 26]. However, HLA genotypes did not predict PTDM in our study.

Controversial data are equally documented in relation to donor transplant, which may be attributed to differences in immunosuppressive regimen and demographic variables. Therefore, DD transplant may be a risk PTDM when compared to LD transplant [17], while other studies, including our study, did not find that association [18, 27].

Early glucose metabolism impairment after transplant, such as altered fasting glucose during the first week after transplant, may also predict a higher risk of developing PTDM [27]. Although glycated hemoglobin (HbA_1c_), measured within the first three months after transplant, does not predict accurately PTDM risk, values at 90 days are strong predictor of PTDM at 1-year and 3-year posttransplant [28]. Thus, impaired insulin secretion appears to be the predominant pathophysiological feature after kidney transplant, and early therapeutic interventions that preserve, maintain, or improve *β*-cell function are potentially beneficial in this population [29].

Metabolic adverse effects of immunosuppressive drugs include all drugs, such as corticosteroids, calcineurin inhibitors, and mTORi. All recipients used corticosteroids in our study. Their diabetogenic effects are attributable to either direct events (increase in insulin resistance associated to higher rates of gluconeogenesis in the liver) or indirect events (weight gain, hyperphagia, the increase in lipolysis-induced dyslipidemia, and the reduction in muscle mass, in glucose uptake, and in glycogen synthesis in skeletal muscle cells) [7]. However, withdrawal of 5 mg prednisolone may not modify significantly insulin sensitivity [30]. In addition, there is a significant risk of acute rejection after corticosteroid withdrawal, as well as worsening of proteinuria and glomerulonephritis recurrence [31, 32]. In our cohort, cumulative corticosteroid dose did not increase the risk of PTDM, yet mean dose was slightly higher in the PTDM (+) group.

Calcineurin inhibitors are also a risk factor for PTDM, mainly FK-based regimen [17, 28, 33]. We documented that FK trough level, even at therapeutic target, within 1-, 3-, and 6-month posttransplant was predictors of PTDM, as reported in liver transplant for FK trough levels greater than 5.9 ng/mL in the sixth month [34] and after pancreas transplant, when high trough levels of FK lead to islet cell swelling and vacuolization [35]. FK-mediated diabetogenic effects, such as *β*-cell apoptosis, decrease in insulin exocytosis, and reduction in insulin gene transcription [36], may explain these findings. Therefore, tailoring FK trough level may improve pancreatic *β*-cell function, as shown by increased C-peptide and insulin secretion [37].

Sirolimus may also cause glucose intolerance, hyperinsulinemia, and hypertriglyceridemia, which is due to increased hepatic gluconeogenesis and reduced stimulated glucose uptake in skeletal muscle [8, 38, 39]. To note, everolimus seems to have less impact on islet cell function when compared to sirolimus [8]. Despite decreasing PTDM incidence at 30 months, when everolimus was compared to CsA, higher rates of rejection were found [40], and others [41] verified no benefit on PTDM incidence with early conversion. Due to the low number of patients under mTORi in our study (<10%), we did not find an association between these drugs and PTDM.

Increased plasma TG and decreased HDL concentration are hallmarks of dyslipidemia in individuals with insulin resistance and may strongly predict cardiovascular events [42]. Values of TG/HDL > 2.2 were associated with an increase in atherogenic lipid phenotype [43]. Annual incidence of DM increases 2-fold when TG/HDL ratio is higher than 3.5 and may predict coronary heart disease, cardiovascular disease mortality, and metabolic syndrome in men [14]. Likewise, increased TG/HDL ratio was related to worsening glucose homeostasis, poor glycemic control, and prevalent microangiopathy complications in women with type 2 DM [44]. In our study, a TG/HDL ratio ≥ 3.5 at 1 year and 3 years after transplant was a major risk factor for PTDM diagnosis and maintenance over time, so that more than 50% of individuals in the PTDM (+) group exhibited that value. Therefore, our findings indicate that higher ratios of TG/HDL posttransplant may be used as surrogate marker of insulin resistance and, ultimately, to PTDM.

In line of these findings, we verified that transient hyperglycaemia within the first three months after transplant was also a predictor of PTDM. Although hyperglycaemia is very common in the early posttransplant period (~90%) due to several conditions, such as immunosuppressive regimen adverse events, rejection therapy, and infection [13], its impact on PTDM requires further analyses in large studies. Therefore, early risk factors for PTDM found in our study, such as recipient age, pretransplant hyperglycaemia, pretransplant BMI > 25 kg/m^2^, and transient hyperglycaemia, indicate that these patients might have insulin resistance for a long time and/or a decreased pancreatic reserve associated with insulin secretion deficiency. Additionally, overweight is a risk factor for insulin resistance. Excess of fat leads to a chronic inflammation state that is associated with macrophage recruitment to adipocytes and release of proinflammatory adipokines, which, ultimately, downregulates insulin signaling and results in PTDM [45–47]. Of importance, we observed an increase in weight (~10%) within three years after transplant in all patients, independently of PTDM diagnosis. Yet, absolute weight was higher in the PTDM (+) group since the pretransplant period and remained higher throughout all period, as also described by others [18]. That finding supports lifestyle modification and nutritional approaches in all kidney transplant candidates that exhibit increased BMI before transplant. Tacrolimus-associated diabetogenic effects combined to a persistent status of insulin resistance, as assessed by TG/HDL ratio, may contribute equally to PTDM and, therefore, may be useful for risk stratification of patients to determine appropriate strategies to reduce PTDM development.

Almost 80% of our patients exhibited hypertension, independently of PTDM development. Although *β*-blockers are associated with insulin resistance and insulin secretion reduction, and therefore, type 2 DM and PTDM [9, 48], we did not find that association. However, 71% of our patients who developed PTDM were under calcium channel blockers (CCBs), mainly amlodipine, as opposed to 54% of those who did not develop PTDM. Although CCBs are generally considered as having an overall neutral metabolic profile, there is evidence that CCBs are associated with higher levels of insulin resistance, as assessed by HOMA (Homeostasis Model Assessment), when compared to ARBs and ACEIs, and a lower incidence compared to *β*-blockers and diuretics [48]. However, amlodipine and enalapril demonstrated to have similar effect on insulin sensitivity using euglycemic hyperinsulinemic clamp in patients with mild to moderate hypertension [49]. Therefore, the conflicting effect of CCBs on glucose metabolism may be explained by the fact that these drugs differ in their inhibitory capacity on N-type and L-type calcium channels and the release of norepinephrine from the sympathetic nerve ending [50]. Accordingly, compared with amlodipine administration, azelnidipine significantly decreased levels of glucose and insulin 120 min after oral glucose tolerance test [51], although HOMA index was not different with amlodipine, manidipine, and cilnidipine [52]. Thus, further studies are required to assess the impact of CCBs on PTDM.

Other emerging risk factors for PTDM include genetic factors, such as leptin receptor [53] and cytochrome CY2224A1 [54] gene polymorphisms, and require further studies to be evaluated in the light of immunosuppressive regimen and other risk factors. Likewise, the identification of inflammation-related biomarkers and the risk of PTDM entail novel perspectives for early detection and treatment of that disease [55].

Of importance, lifestyle intervention, tight glycemic control, early introduction of ACEIs or ARBs, and tailoring immunosuppressive regimen may mitigate PTDM-associated complications in kidney transplant setting [56, 57]. Therefore, PTDM is a condition not only to be aware of but also to treat, such as using metformin [58, 59], meglitinides, GLP1 agonists, DPP4 inhibitors, and SGLT2 inhibitors [5]. Likewise, early use of basal insulin may significantly reduce the chances of PTDM, possibly by insulin-mediated *β*-cell protection and “resting” [60].

Our study has some limitations, such as the lack of identification of metabolic syndrome over time, and no measurement of proteinuria and other glucose parameters, such as HbA_1c_ and HOMA index throughout the study as well. Furthermore, the short follow-up did not allow us to verify the impact of PTDM on eGFR over time. Of importance, an educational programme based on lectures and individual planning with focus on lifestyle modifications (diet, exercise, weight control, and smoking cessation) and early identification of metabolic syndrome were established at our transplant center.

In conclusion, our findings indicate that major risks for PTDM development were modifiable variables. We identified a temporal distribution of these variables. Early risk factors included acute rejection, transient hyperglycaemia, and higher trough levels of FK in patients who already presented higher values of pretransplant BMI and hyperglycaemia. Later risk factor included the increase in insulin resistance, as assessed by TG/HDL ratio, which contributed to PTDM maintenance. Therefore, identification of these risk factors supports patient risk stratification to determine appropriate strategies for risk reduction in PTDM in kidney transplant setting. Our study contributes to set the basis for further studies comprising larger cohorts in multicenter studies.

## Figures and Tables

**Table 1:** Demographic data (*n* = 450).

Variable		*N* (%)
Gender	Male; female	270 (60%); 180 (40%)

Age (years)	18-39	186 (41.3%)
	40-59	240 (53.3%)
	≥59	24 (5.3%)

Ethnicity	Caucasian; non-Caucasian	238 (52.8%); 212 (47.2%)

CKD etiology	Hypertension	67 (14.8%)
	Glomerulonephritis	93 (20.6%)
	ADPKD	43 (9.5%)
	Lupus	14 (3.1%)
	Other	40 (8.9%)
	Unknown	193 (42.9%)

RRT	Yes; no	417 (92.6%); 33 (7.4%)

Donor	Living; deceased	190 (42.2%); 260 (57.8%)

Pretransplant BMI (kg/m^2^)	<18	33 (7.3%)
	18-24.9	274 (60.8%)
	25-29.9	109 (24.2%)
	≥30	34 (7.5%)

BMI: body mass index; ADPKD: autosomal-dominant polycystic kidney disease; RRT: renal replacement therapy.

**Table 2:** Modifiable and nonmodifiable risk factors for developing PTDM including nontransplant-related variables.

Nontransplant variables						
	PTDM (+) (*n* = 61)	PTDM (-) (*n* = 389)	*P*	OR	95% CI	
					Inferior	Upper
Recipient age (years)	46.2 ± 1.3	40.7 ± 0.6	0.001	1.04	1.02	1.07
Male (%, *n*)	62.3% (38)	51.5% (232)	0.69	0.89	0.51	1.56
Non-black (%, *n*)	52.5% (32)	52.9% (206)	0.94	0.98	0.57	1.68
RRT (%, *n*)	96.7% (59)	92% (358)	0.21	0.39	0.09	1.68
Mean time on dialysis (months)	44.6	45.1	0.21	0.99	0.99	1.00
ADPKD (%, *n*)	11.5% (7)	9% (35)	0.54	0.76	0.32	1.80
Pretransplant hyperglycaemia (%, *n*)	36% (22)	23.1% (90)	0.032	0.53	0.30	0.95
Living donor (%, *n*)	45.9% (28)	41.6% (162)	0.53	0.84	0.49	1.45
Positive CMV serology (%, *n*)	85.2% (52)	87.9% (342)	0.52	1.29	0.56	2.79
BMI ≥ 25 kg/m^2^ (%, *n*)	57.4% (35)	27.7% (108)	0.0001	3.50	2.01	6.09
HLA DR3 (%, *n*)	11.5% (7)	20% (78)	0.12	1.93	0.85	4.42
HLA A2 (%, *n*)	57.3% (35)	50.1% (195)	0.29	0.75	0.43	1.29
HLAB35 (%, *n*)	18% (11)	23.6% (92)	0.33	1.41	0.70	2.82
HLADR4 (%, *n*)	27.8% (17)	21.3% (83)	0.26	0.70	0.38	1.29
HLADR7 (%, *n*)	29.5% (18)	22.8% (89)	0.26	0.71	0.39	1.29
HLADR11 (%, *n*)	27.8% (17)	26.4% (103)	0.82	0.93	0.51	1.70
HLADR13 (%, *n*)	32.7% (20)	27% (105)	0.35	0.76	0.42	1.35

**Table 3:** Modifiable and nonmodifiable risk factors for developing PTDM including transplant-related variables.

Transplant-related variables						
	PTDM (+) (*n* = 61)	PTDM (-) (*n* = 389)	*P*	OR	95% CI	
					Inferior	Upper
Acute rejection (%, *n*)	45.9% (28)	30.8% (120)	0.02	0.53	0.30	0.91
Cumulative steroid dose (mg/kg)	70.3 ± 7.7	61.8 ± 1.8	0.14	1.00	0.99	1.01
Tacrolimus (%, *n*)	85.2% (52)	85% (331)	0.97	0.99	0.46	2.11
Mycophenolate of sodium (%, *n*)	44.2% (27)	54.7% (213)	0.13	1.52	0.88	2.62
Cyclosporine (%, *n*)	6.5% (4)	9.2% (36)	0.49	1.45	0.50	4.24
Azathioprine (%, *n*)	49.2% (30)	39.8% (155)	0.17	0.68	0.40	1.18
Sirolimus (%, *n*)	3.2% (2)	3.3% (13)	0.98	1.02	0.22	4.63
Everolimus (%, *n*)	9.8% (6)	6.4% (25)	0.33	0.63	0.25	1.60
CMV infection (%, *n*)	36% (22)	34.7% (135)	0.84	0.94	0.54	1.65
Transient hyperglycaemia (%, n)	86.8% (53)	18.5% (72)	0.0001	0.03	0.02	0.07
Delayed graft function (%, *n*)	31.1% (19)	34.2% (133)	0.64	1.15	0.64	2.05
Hypertension (%, *n*)	86.9% (53)	79.7% (310)	0.19	0.59	0.27	1.30
Calcium channel blockers (%, *n*)	70.5% (43)	53.5% (208)	0.014	0.48	0.27	0.86
*β*-Blockers (%, *n*)	54% (33)	41.9% (163)	0.07	0.61	0.36	1.05
TG/HDL ≥ 3.5 at 6 months	42.6% (26)	32.9% (128)	0.14	0.66	0.38	1.14
TG/HDL ≥ 3.5 at 1 year	55.7% (34)	38% (148)	0.01	0.49	0.28	0.84
TG/HDL ≥ 3.5 at 2 years	41% (25)	32.4% (126)	0.19	0.69	0.40	1.12
TG/HDL ≥ 3.5 at 3 years	52.4% (32)	25.7% (100)	0.0001	0.31	0.18	0.54
FK trough level at 1 month	11.4 ± 4.2	9.8 ± 3.6	0.004	1.11	1.03	1.19
FK trough level at 3 months	9.5 ± 3.8	8.4 ± 3	0.016	1.11	1.02	1.20
FK trough level at 6 months	9.2 ± 3.3	7.9 ± 3	0.009	1.12	1.03	1.22
FK trough level at 1 year	8.1 ± 3.3	7.4 ± 2.8	0.13	1.08	0.979	1.18
FK trough level at 2 years	7.9 ± 2.6	7.3 ± 3.1	0.18	1.06	0.972	1.16
FK trough level at 3 years	7.7 ± 2.7	7.1 ± 2.6	0.15	1.08	0.973	1.19

**Table 4:** Multivariate analyses of risk factors for developing PTDM.

	PTDM (+) (*n* = 61)	PTDM (-) (*n* = 389)	*P*	OR	95% CI	
					Inferior	Upper
Demographic variables						
Recipient age (years)	46.2 ± 1.3	40.7 ± 0.6	0.59	1.00	0.99	1.00
Pretransplant hyperglycaemia (%, *n*)	36% (22)	23.1% (90)	0.035	0.53	0.29	0.96
Pretransplant BMI ≥ 25 kg/m^2^ (%, *n*)	57.4% (35)	27.7% (108)	0.0001	0.28	0.20	0.50
Cardiometabolic variables						
Transient hyperglycaemia (%, *n*)	86.8% (53)	18.5% (72)	0.0001	0.04	0.02	0.08
TG/HDL ≥ 3.5 at 1 year	55.7% (34)	38% (148)	0.794	1.10	0.55	2.2
TG/HDL ≥ 3.5 at 3 years	52.4% (32)	25.7% (100)	0.003	0.35	0.17	0.70

## Data Availability

The data used to support the findings of this study are available from the corresponding author upon request.

## Supplementary Materials

Supplementary Materials Figure 1S: PTDM did not have impact on eGFR (estimated glomerular filtration rate) values in the PTDM (+) group of both living donor (LD) and deceased donor (DD) patients during any time-point up to 36 months (m) after transplant (*P* = 0.26). Figure 2S: in the PTDM (-) group, LD recipients had a higher eGFR when compared to DD recipients until 24 months after transplant (~10 mL/min/1.73 m2), so that in the first month after transplant that difference was more pronounced (~20 mL/min/1.73 m2, *P* < 0.0001). Figure 3S: eGFR values of LD recipients in accordance with PTDM development. These patients exhibited lower eGFR values within 36 months when compared to the first month after transplant in the PTDM (-) group (*P* = 0.007), while LD recipients in the PTDM (+) group did not present difference over time. Figure 4S: eGFR values of DD recipients in accordance with PTDM development among DD recipients, eGFR was higher over time until 24 months after transplant in the PTDM (-) group (*P* < 0.0001). In DD recipients who developed PTDM, eGFR was not affected over time.
