# Supplementary material for: Modifiable Variables Are Major Risk Factors for Posttransplant Diabetes Mellitus in a Time-Dependent Manner in Kidney Transplant: An Observational Cohort Study
Source: J Diabetes Res. 2020 Mar 18;2020:1938703. doi: 10.1155/2020/1938703 (PMC7109550; doi:10.1155/2020/1938703)
Supplement: Supplementary Materials — Figure 1S: PTDM did not have impact on eGFR (estimated glomerular filtration rate) values in the PTDM (+) group of both living donor (LD) and deceased donor (DD) patients during any time-point up to 36 months (m) after transplant (P = 0.26). Figure 2S: in the PTDM (-) group, LD recipients had a higher eGFR when compared to DD recipients until 24 months after transplant (~10 mL/min/1.73 m2), so that in the first month after transplant that difference was more pronounced (~20 mL/min/1.73 m2, P < 0.0001). Figure 3S: eGFR values of LD recipients in accordance with PTDM development. These patients exhibited lower eGFR values within 36 months when compared to the first month after transplant in the PTDM (-) group (P = 0.007), while LD recipients in the PTDM (+) group did not present difference over time. Figure 4S: eGFR values of DD recipients in accordance with PTDM development among DD recipients, eGFR was higher over time until 24 months after transplant in the PTDM (-) group (P < 0.0001). In DD recipients who developed PTDM, eGFR was not affected over time. [file 1938703.f1.doc]

**Modifiable variables are major risk factors for post-transplant diabetes mellitus in a time-dependent manner in kidney transplant: an observational cohort study**

Débora Dias de Lucena^1^, João Roberto de Sá^2^, José O Medina-Pestana^1^, Érika Bevilaqua Rangel^1,3*^

^1^Nephrology Division, Universidade Federal de São Paulo/Hospital do Rim, São Paulo, SP, Brazil

^2^Endocrinology Division, Universidade Federal de São Paulo, São Paulo, SP, Brazil

^3^Hospital Israelita Albert Einstein, São Paulo, SP, Brazil

**Corresponding Author:**

* Érika B Rangel, MD, PhD

Address: Nephrology Division, Federal University of São Paulo, 740 Botucatu Street, Vila Clementino, 04023-900, São Paulo, SP, Brazil

E-mail: [erikabr@uol.com.br](mailto:erikabr@uol.com.br)

**Figure 1S**. PTDM did not have impact on eGFR (estimated glomerular filtration rate) values in the PTDM (+) group of both living donor (LD) and deceased donor (DD) patients during any time-point up to 36 months (m) after transplant (*P*=0.26).

**Figure 2S**. In the PTDM (-) group, LD recipients had a higher eGFR when compared to DD recipients until 24 months after transplant (~10ml/min/1.73m^2^), so that in the first month after transplant that difference was more pronounced (~20ml/min/1.73m^2^, *P*<0.0001).

**Figure 3S**. eGFR values of LD recipients in accordance with PTDM development. These patients exhibited lower eGFR values within 36 months when compared to the first month after transplant in the PTDM (-) group (*P*=0.007), while LD recipients in the PTDM (+) group did not present difference over time.

**Figure 4S**. . eGFR values of DD recipients in accordance with PTDM development Among DD recipients, eGFR was higher over time until 24 months after transplant in PTDM (-) group (*P*<0.0001). In DD recipients who developed PTDM, eGFR was not affected over time.
